# DUFs: families in search of function

**DOI:** 10.1107/S1744309110001685

**Published:** 2010-03-05

**Authors:** Alex Bateman, Penny Coggill, Robert D. Finn

**Affiliations:** a Wellcome Trust Sanger Institute, Wellcome Trust Genome Campus, Hinxton, Cambridgeshire CB10 1SA, England

**Keywords:** structural genomics, domain of unknown function (DUF), uncharacterized protein family (UPF), Pfam

## Abstract

Domains of unknown function (DUFs) are a large set of uncharacterized protein families that are found in the Pfam database. Here, the scale and growth of functionally uncharacterized families in biological databases are surveyed and the prospects for discovering their function are examined. In particular, the important role that structural genomics can play in identifying potential function is evaluated.

## Abstract 2

Domains of unknown function (DUFs) are a large set of uncharacterized protein families that structural genomics is helping biologists to understand functionally.

## 1. Introduction

To achieve the ultimate goal of systems biology to model both living cells and organisms, we must know the functions of all their con­stituent parts. Even for the most intensively experimentally studied organisms there are many proteins for which we have no clue as to their function. For example, in the yeast *Saccharomyces cerevisiae* approximately 1000 proteins (17% of the genome) are still uncharacterized (Pena-Castillo & Hughes, 2007 ▶).

The Pfam database is a collection of protein families and domains that has been widely used for annotating sequenced genomes (Finn *et al.*, 2008 ▶). Grouping each protein encoded by a genome into a family of homologous proteins can help to annotate its function. For example, if one or more members of a Pfam family have an experimentally determined function then this function can be tentatively assigned to the other proteins in that family. Using this approach, the majority of proteins encoded by a genome can be annotated despite the fact that not a single protein in that particular genome has ever been experimentally investigated. Even in the absence of functional information, grouping proteins into families can indicate those amino acids within the proteins that are conserved and hence are potentially functionally important. Approximately three-quarters of all known proteins now match one or another of the 10 000 protein families in Pfam (Finn *et al.*, 2008 ▶).

**Figure 1:**
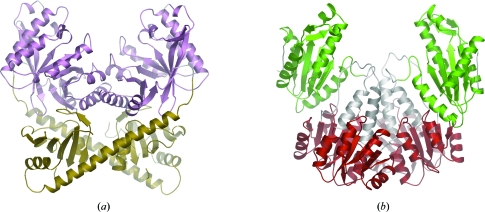
(*a*) The EAL domain (PF00563, magenta) from PDB entry 2bas (Minasov *et al.*, 2009 ▶), formerly known as DUF2, is now known to function as a cyclic diguanylate-specific phosphodiesterase enzyme. The structure of the associated C-terminal domain YkuI_C (PF10388) is coloured gold. (*b*) The GGDEF domain (in green) from PDB entry 1w25 (Chan *et al.*, 2004 ▶), formerly known as DUF1, is now known to function as a diguanylate cyclase enzyme. There are two copies of the response regulator receiver domain (PF00072, red) found at the N-terminus of each monomer within the dimeric structure. Figs. 1 and 4 were produced using *OpenAstexViewer* 3.0 (Hartshorn, 2002 ▶).

Domains of unknown function, or DUFs, are a large set of families within the Pfam database that do not include any protein of known function. Although called DUFs, for many of these families it is not known whether they actually represent one protein domain or many. The DUF naming scheme was introduced by Chris Ponting through the addition of DUF1 and DUF2 to the SMART database (Schultz *et al.*, 1998 ▶). These two domains were found to be widely distributed in bacterial signalling proteins. Subsequently, the functions of these domains were identified and they have since been renamed as the GGDEF (PF00990, SMART accession SM00267) and EAL (PF00563, SM00052) domains, respectively. The structures of these two proteins domains are shown in Figs. 1 ▶(*a*) and 1 ▶(*b*). Both of these domains are involved in processing cyclic diguanylate, a universal bacterial second-messenger molecule (Romling & Simm, 2009 ▶). Although no further DUFs appeared in SMART, DUF1 and DUF2 were added to Pfam in 1997 and little did Chris Ponting realise that he was starting a trend that would see thousands of uncharacterized and largely anonymous families being added to the protein-family databases.

DUFs are created with the same care and attention as all other Pfam families. The only difference is that the curators are unable to identify any functional information from the scientific literature at the time that they are carrying out their analysis.

## 2. The scale of uncharacterized families

**Figure 2:**
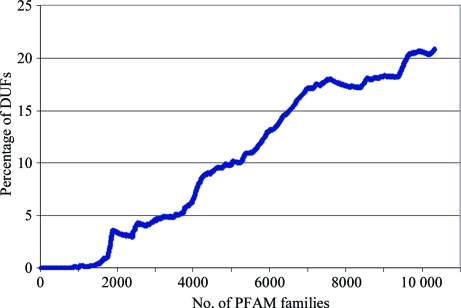
A graph showing the growth of DUFs as a percentage of all families added to Pfam at the time of release 23.0.

In Pfam release 23.0, the DUF numbering scheme reached DUF2607 and the fraction of DUF families in Pfam had increased to about 22% of all families (Fig. 2 ▶). The number of DUFs is on the increase for three reasons: (i) Pfam already contains most of the large functionally well characterized families, (ii) DUFs require little annotation and so are often easy families to add to Pfam and (iii) the large number of new genomic and metagenomic sequences allows the description of many new clade-specific families. We expect that the number of DUFs will soon outnumber the families with known function being added to Pfam. Before panicking about being inundated with DUFs, it is worth noting that these families are becoming progressively smaller and so their contribution, by number of sequences, is not too large (Sammut *et al.*, 2008 ▶).

The UniProt database also contains a series of uncharacterized protein families called UPFs (Doerks *et al.*, 1998 ▶). The UPF series had reached UPF0747, but 136 of these have now been assigned a known function. A full list of UPFs can be found at http://www.uniprot.org/docs/upflist. In some cases, the same protein family is identified as a DUF (in Pfam) and a UPF (in UniProt). However, in most cases, the Pfam DUF family has many more members than the related UPF family.

**Figure 3:**
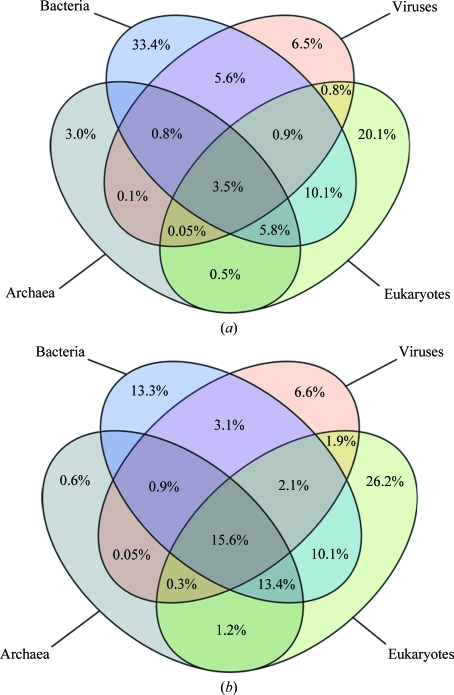
Venn diagrams showing (*a*) the distribution of DUF families (including Pfam UPFs) in different kingdoms and (*b*) the distribution of all other Pfam families in different kingdoms.

In Fig. 3 ▶ we examine the distribution of DUF and non-DUF families in different species. Just over one-third of DUF families are restricted to the eubacterial kingdom as a consequence of the very large number of prokaryotic genomes that have now been sequenced. The species distribution shows that 20% of the DUFs are restricted to eukaryotes but that only 3% are found only in archaebacteria. Compared with the 15% of non-DUF families that are found in all kingdoms of life, only 3.5% of DUF families are found in all kingdoms. It is interesting to note the significant presence of DUFs even within larger systems of known biology. For example, around 40% of the genes identified in *Bacteroides thetaiotaomicron* polysaccharide-utilization loci (PULs) are not homologous to any genes of known function (Martens *et al.*, 2009 ▶). This suggests that DUF families are more likely to represent biological functions that are specific to certain individuals, groups of organisms or environmental conditions rather than being part of the core machinery common to all life.

It should also be noted that many of these families have been given a more descriptive name within Pfam and other family databases yet are still functionally uncharacterized. For example, the YukD family (PF08817) is named after the *B. subtilis* protein that adopts a ubiquitin-like fold but its function is still unknown (van den Ent & Lowe, 2005 ▶). Thus, consideration of just DUF and UPF families underestimates the actual fraction of uncharacterized families in these databases.

## 3. Finding function

It is sometimes surprisingly difficult to determine the specific function of a protein. In some cases, identifying a nucleotide-binding P-loop motif might be considered to be sufficient to define a function for that protein. However, knowing that a protein binds a nucleotide does not tell us what biological process the protein is participating in or what action or role it might be carrying out.

Proteins of known function can also contain DUFs. For example, the very well characterized Dicer endonuclease contains a domain first named DUF283 (PF03368). The strong sequence conservation of this domain within Dicer proteins indicated that it was likely to convey an important function, yet at the time of curation this region was uncharacterized. Subsequently, it has been found that DUF283 shows sequence similarity to double-stranded RNA-binding domains, which indeed represents a highly likely function for a domain within the Dicer dsRNA endonuclease (Dlakic, 2006 ▶).

Identifying functions for DUFs is extremely important for characterizing lists of biological parts. Essentially, there are three ways to determine the function of an uncharacterized domain: the first involves identifying similarity to a domain of known function, either by sequence comparison or by structural analysis of a newly solved structure of one of the member proteins, the second involves using contextual information such as genomic context to computationally identify function, as employed by databases such as STRING (Jensen *et al.*, 2009 ▶) and PROLINKS (Bowers *et al.*, 2004 ▶), and the third is through good old-fashioned molecular biology or biochemistry. Notably, Sir Rich Roberts put forward a proposal to stimulate experimentation on such uncharacterized proteins (Roberts, 2004 ▶) and there have been commendable attempts to functionally characterize proteins on a large scale (Martzen *et al.*, 1999 ▶). Martzen and coworkers identified that DUF27 (PF01661) may possess an adenosine phosphate-ribose 1′-phosphate processing activity. This activity was subsequently experimentally confirmed and this domain is now called the MACRO domain (Karras *et al.*, 2005 ▶). One issue with identifying the functions of proteins classified as DUFs is that they are usually non-essential. A systematic knockout screen of *B. subtilis* has indicated that only 4% of essential genes have unknown function (Kobayashi *et al.*, 2003 ▶). These results imply that the knockout strategies that are routinely employed to identify a phenotype to help understand function are much less likely to be fruitful for identification of the function of DUFs.

Slowly, momentum is being gained and more functions for DUFs are being identified. Since we began adding DUFs to Pfam nearly ten years ago more than 270 of them have been renamed or reclassified, usually when a function has been identified. Pfam curators have not yet had time to systematically recheck all of the existing 2000+ DUFs to see whether new functional information for either the family or the individual protein has been identified. However, over the coming year we hope to revisit all of them and rename and re-annotate those where function is now known. This exercise should potentially identify 100+ families that have now been characterized. We ask users that if they know of any recently identified functions for these families they please contact the authors of the Pfam database.

Many of the DUF families had a rather limited membership when added to Pfam. As additional sequences are incorporated into the sequence database and added to the relevant families, we sometimes determine that these families are actually subfamilies of much larger families. In such cases, the DUF subfamily is merged into the larger parent family. Just under 200 such merges have occurred after successive sequence inclusions.

Various tools are now available that can help to identify relationships between DUFs and other functionally characterized families. Profile-HMM comparison tools, such as *HHsearch* (Söding, 2005 ▶), *PRC* (Madera, 2008 ▶), *SIMPRO* (Jung & Kim, 2009 ▶) and *SCOOP* (Bateman & Finn, 2007 ▶), have proved to be very useful in this regard. In many cases, these programs can identify distant yet functionally relevant similarities that standard sequence and profile methods may miss. When these similarities are identified, it is possible to merge the two families into one large but more divergent single family. More often than not in such cases a single profile HMM is not sensitive enough to detect all the members of two or more distantly related families. When we are confident that two or more families are derived from a common evolutionary ancestor, we group them together in Pfam clans. Pfam clans are collections of families that are thought to have originated from a common evolutionary ancestor. As of Pfam release 23.0, 199 DUFs belong to clans in which there are one or more related families with known function. These distant relationships of DUFs with non-DUFs within a clan can also provide clues to the likely function of DUFs, but one must be especially cautious when transferring function.

## 4. The contribution of structural genomics

In recent years, structural genomics initiatives have solved the structures of literally hundreds of proteins within uncharacterized families. In many cases, this has helped to narrow down the possible function of a family (Jaroszewski *et al.*, 2009 ▶). Protein structures can help to identify protein function in a number of different ways.

### 4.1. Cocrystallization of a ligand

In an accompanying paper in this issue on the structure of the DUF2006 family (Chiu *et al.*, 2010 ▶), the authors identified that this family contains two structurally similar domains that belong to the calycin superfamily (Flower, 1993 ▶). While the function of DUF2006 (PF09410) remains unknown, the calycin superfamily includes a wide variety of families with known binding functions, such as the lipocalins. Although this family does not possess a large cavity like the lipocalins, it does contain a smaller cavity in the N-terminal domain that harbours a glycerol molecule and is suggestive of a ligand-binding site. Glycerol was part of the crystallization solution and does not represent the physiological ligand.

**Figure 4:**
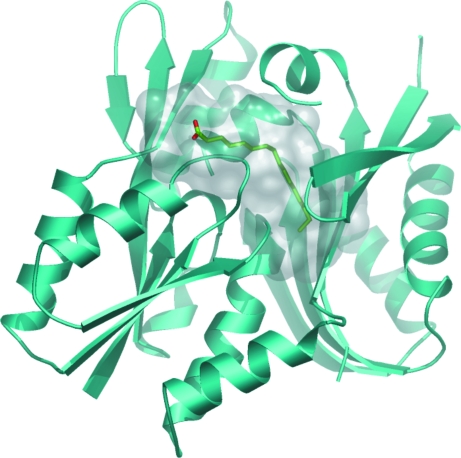
TM841 protein binding to palmitate (represented as sticks) from PDB entry 1mgp. The protein–ligand interaction interface is represented as a semi-transparent grey surface.

Unequivocal evidence for the function of a protein can sometimes be found when the physiological ligand is cocrystallized with the protein. The structure of *Thermotoga maritima* protein TM841, a protein from the family formerly called DUF194 (renamed DegV; PF02645), has been solved (Schulze-Gahmen *et al.*, 2003 ▶). This protein contained a bound palmitate fatty-acid molecule (Fig. 4 ▶). However, the protein has no conserved residues lining the binding pocket that are likely to be catalytic, suggesting that this protein has a lipid-transport function.

### 4.2. Identify distant functional relationships

Relationships between protein structures can be found even when similarities are not detectable with the most sensitive sequence-comparison methods. This implies that solution of the structure of an uncharacterized protein can identify relationships with functionally characterized proteins. In such a case, the two proteins will only be very distantly related, so any assumption of a common function must be tentative. However, even a hint of similarity can greatly aid the process of designing experiments to determine the function of a DUF. For example, DUF442 (PF04273) was shown to be a non­classical phosphatase enzyme based on its structural similarity to known enzymes (Krishna *et al.*, 2007 ▶) and so is grouped with families of the phosphatase clan (CL0031). Partial structural similarities combined with bioinformatic analysis can also provide clues to function. Several such examples can be found in this issue, many of which indicate functions relating to particular environmental conditions, such as stress or pathogenesis (Bakolitsa, Bateman *et al.*, 2010 ▶; Bakolitsa, Kumar *et al.*, 2010 ▶), or particular features of the environment (Miller *et al.*, 2010 ▶).

### 4.3. Identify and improve domain boundaries

In another paper in this issue describing the structure of the DUF1470 (PF07336) family (Bakolitsa, Bateman *et al.*, 2010 ▶), it was discovered that the DUF was composed of two distinct protein domains: a novel domain called the ABATE domain at the N-­terminus and a treble-clef-like zinc finger called a CGNR finger (PF11706; release 24.0) at the C-terminus. By splitting the original longer protein into its constituent domains, further homologues were detected. Similarly, structure determination of the first representative of DUF0035 (PF01796) revealed a zinc ribbon and an OB-fold domain (Krishna *et al.*, 2010 ▶), allowing the signature for this family to be split into two new entries.

## 5. Conclusions

Entirely new biological systems and pathways are likely to still be waiting to be discovered. Even ten years ago we did not know of the existence of microRNAs or their associated protein machinery. Given the amazing potential of microRNAs to regulate gene expression, it seems likely that we are still missing important and large biological systems. DUFs remain a treasure trove of novel biology waiting to be plundered. Structural genomics has provided a wealth of data for many of these families. With detailed and careful analysis, these structures can give strong hints as to their likely functions. Unfortunately, a curious consequence of the tremendous success of the structural genomics initiatives, in particular the PSI, means that the ability to produce structures has outstripped our ability to analyze them in detail. Therefore, it would seem that there are great opportunities for present-day pioneers to characterize DUFs both com­putationally and experimentally.
